# Household Food Insufficiency and Chronic Pain among Children in the US: A National Study

**DOI:** 10.3390/children10020185

**Published:** 2023-01-19

**Authors:** See Wan Tham, Emily F. Law, Tonya M. Palermo, Flavia P. Kapos, Jason A. Mendoza, Cornelius B. Groenewald

**Affiliations:** 1 Department of Anesthesiology and Pain Medicine, University of Washington School of Medicine, Seattle, WA 98195, USA; 2 Center for Child Health, Behavior and Development, Seattle Children’s Research Institute, Seattle, WA 98101, USA; 3 Department of Pediatrics, University of Washington School of Medicine, Seattle, WA 98195, USA; 4 Public Health Sciences Division, Fred Hutchinson Cancer Research Center, Seattle, WA 98109, USA

**Keywords:** chronic pain, food insufficiency, pediatrics, National Survey of Children’s Health

## Abstract

This study aimed to determine the prevalence of pediatric chronic pain by household food sufficiency status and examine whether food insufficiency would be associated with greater risk for chronic pain. We analyzed data from the 2019–2020 National Survey of Children’s Health of 48,410 children (6–17 years) in the United States. Across the sample, 26.1% (95% CI: 25.2–27.0) experienced mild food insufficiency and 5.1% (95% CI: 4.6–5.7) moderate/severe food insufficiency. The prevalence of chronic pain was higher among children with mild (13.7%) and moderate/severe food insufficiency (20.6%) relative to children in food-sufficient households (6.7%, *p* < 0.001). After adjusting for a priori covariates (individual: age, sex, race/ethnicity, anxiety, depression, other health conditions, adverse childhood events; household: poverty, parent education, physical and mental health; community: region of residence), multivariable logistic regression revealed that children with mild food insufficiency had 1.6 times greater odds of having chronic pain (95% CI: 1.4–1.9, *p* < 0.0001) and those with moderate/severe food insufficiency, 1.9 higher odds (95% CI: 1.4–2.7, *p* < 0.0001) relative to food-sufficient children. The dose–response relationship between food insufficiency and childhood chronic pain highlights the importance of further research to identify underlying mechanisms and evaluate the impact of food insufficiency on the onset and persistence of chronic pain across the lifespan.

## 1. Introduction

Chronic pain is a significant public health concern, affecting up to 45% of children in the United States (US) [1,2]. During childhood, chronic pain may be related to health conditions such as sickle cell disease, cancer, and arthritis. However, a significant number of children have primary chronic pain conditions, the most common of which are headaches, abdominal pain, and musculoskeletal pain [3]. Childhood chronic pain can be associated with significant physical, mental, and behavioral health concerns, including depression, anxiety, and sleep disturbance. Moreover, children with chronic pain often have significant functional limitations, including reduced activity participation, poorer academic performance, and peer relationship deficiencies [4,5]. Furthermore, the economic burden of pediatric chronic pain is estimated at USD 19.5 billion annually, exceeding expenditures on highly prevalent pediatric conditions, such as asthma and attention-deficit and hyperactivity disorders [6]. Given the significant public health impact, it is urgent that we identify modifiable risk factors of childhood chronic pain.

There has been a steady focus on characterizing the biopsychosocial risk factors associated with pediatric chronic pain. However, research has been more limited in examining social risk factors of pediatric chronic pain [7]. To date, several factors have been identified, including the influence of race, ethnicity, and socioeconomic status. For example, children who identified as belonging to an underrepresented racial and/or ethnic group [8,9], and who resided in households and neighborhoods with lower socioeconomic status were more likely to experience chronic pain [3,10]. More recently, food insecurity has emerged as a consideration for individuals with chronic pain.

Food insecurity, defined as “a lack of consistent access to enough food for an active, healthy lifestyle” is a significant social risk factor of health affecting 6.1 million children in the US [11]. Food insecurity captures a broad concept, indicating limited availability of nutritionally adequate foods, reduced access to food, and/or inadequate quantity of food intake. In the setting where there is not enough food in the household to eat, the term food insufficiency is used to describe this greater severity of food scarcity, often used as a proxy for “hunger” [12,13,14,15]. Since 2015, the American Academy of Pediatrics (AAP) has recommended practice and systems levels efforts to mitigate food insecurity given its negative impact on health outcomes [16]. Specifically, food insecurity is associated with increased rates of recurrent respiratory infections, obesity, depression, sleep disturbance, and greater healthcare utilization [17,18,19,20,21]. However, little research has examined the relationship between food insecurity, food insufficiency, and chronic pain. To our knowledge, there is only one pediatric study which found that school-aged children who experienced food insufficiency were more likely to report frequent headaches and stomachaches compared to children who were food-sufficient [22]. Several studies in adult populations have shown that those who experienced food insecurity also had higher prevalence of migraines, musculoskeletal, chest, and abdominal pain, and greater pain-related disability [23,24,25,26,27,28,29]. Moreover, over half of adults who utilized food banks have been found to have a chronic pain diagnosis [30]. There are several hypotheses underlying the complex linkage between food insecurity and chronic pain. At the individual level, poor nutritional patterns may predispose to pain symptom such as headaches. In the household, food insecurity may be a competing priority relative to medical treatment, and untreated pain may increase the risk for the development of chronic pain. Community-level influences, such as residing in more rural areas, may be associated with limited access to healthcare services to evaluate and treat pain. Despite this, links between food insecurity/food insufficiency and chronic pain remain inadequately described in children.

Therefore, the primary aim of this study was to determine the prevalence of chronic pain by household food insufficiency status in a nationally representative sample of children from 6 to 17 years of age in the US. We hypothesized that household food insufficiency would be associated with increased prevalence rates of chronic pain in a dose–effect relationship, with higher prevalence of chronic pain among children with greater severity of food insufficiency. Additionally, we aimed to estimate the adjusted association between food insufficiency and pediatric chronic pain, and hypothesized that food insufficiency would be independently associated with chronic pain status, after controlling for individual, household, and community-level factors.

## 2. Materials and Methods

### 2.1. Study Design and Participants

This study was a secondary analysis of cross-sectional data from the 2019 and 2020 National Survey of Children’s Health (NSCH) available at https://www.childhealthdata.org, accessed on 22 July 2020. Funded and directed by the Maternal and Child Health Bureau in partnership with the Census Bureau and the Centers for Disease Control, the NSCH is conducted annually to provide data on the physical and mental health, and determinants of health of children (0–17 years of age) in the US. Households are randomly sampled via a multistage process, with one child being chosen to be the subject of the survey. Survey data are weighted to represent the national population of civilian, noninstitutionalized children in the US. Parents were the primary respondents, with surveys conducted either via in-person interviews or via web-based surveys (based on preference). The total sample for 2019 and 2020 included 72,210 participants. We excluded those with incomplete data on any of our measures (n = 4871) and children 0–5 years of age (18,929) leaving a final sample of 48,410 children for analysis. We followed the Strengthening the Reporting of Observational Studies in Epidemiology (STROBE) reporting guidelines for cross-sectional studies. Our institutional review board determined that approval was not required, because the study did not involve human subjects (NCHS contains no protected health information and is publicly available).

### 2.2. Measures

#### 2.2.1. Chronic Pain

Parents were asked “During the past 12 months, has this child had frequent or chronic difficulty with repeated or chronic physical pain, including headaches or other back or body pain?”. Response options were “yes” or “no”. Children whose parents responded “yes” were assigned to the chronic pain group. This item has been used to classify chronic pain status in previous studies from NSCH [31].

#### 2.2.2. Food Insufficiency

Parents responded to the following item: “Which of the following statements best describes your household’s ability to afford food you need during the past 12 months?”. There were four response options: “(1) We could always afford to eat nutritious meals; (2) We could always afford to eat enough but not always the kinds of food we should eat; (3) Sometimes we could not afford enough to eat; and (4) Often we could not afford enough to eat”. Consistent with previous studies using data from the NSCH to measure food sufficiency [32,33,34], respondents who chose option 1 were classified as food-sufficient, those who chose option 2 were classified as having mild food insufficiency, while those who chose either option 3 or 4 were classified as moderate/severe food-insufficient.

#### 2.2.3. Covariates

To select covariates for inclusion in multivariable models, we were informed by Weiser et al.’s [35] conceptual framework which specifies three levels of determinants (individual, household, and community) that impact the bidirectional effect of food insufficiency on child health outcomes. We included the following factors: (1) individual (age, sex, race, and ethnicity, child’s chronic health conditions, child’s mental health conditions (anxiety, depression), and history of adverse childhood experiences (ACEs); (2) household (parent mental health, parent physical health, household poverty category, and parent education), and (3) community (geographical region of residence).

##### Individual Factors

Parents reported on their child’s demographic characteristics: age (categorized by us as 6–11 years and 12–17 years), biological sex at birth (male versus female), and race/ethnicity. In the NSCH, response options for race/ethnicity were Hispanic; non-Hispanic Asian; non-Hispanic Black or African American; non-Hispanic other race; non-Hispanic White.

Anxiety and depression were included as separate covariates in analyses, given the high comorbidity among youth with chronic pain [36,37]. Parents were asked whether their child currently had anxiety problems and depression. For analyses, we assigned participants to one of two categories (yes/no), representing whether they currently had anxiety or depression.

Parents reported on their child’s health status: whether their child had been diagnosed with common chronic conditions of childhood as assessed in the NSCH, including asthma, attention-deficit and hyperactivity disorder, allergies, autism, diabetes mellitus, neurodevelopmental delay, arthritis, cystic fibrosis, seizures, and heart conditions. For analyses, we assigned participants to one of two categories (yes/no), representing whether they had any of the chronic health conditions.

Data were collected for adverse childhood events (ACEs). Parents reported on whether their child had ever experienced any of the following nine ACEs: (1) divorce or separation of parents; (2) incarceration of parent; (3) witnessing of domestic violence; (4) cohabitated with someone who is mentally ill and/or suicidal; (5) cohabitated with someone with substance abuse problems; (6) treated or judged unfairly due to their race/ethnicity; (7) experienced the death of a parent; (8) victim of or witnessed violence within their neighborhood; and (9) hardship suffered because low income. All ACEs were categorized as binary (yes/no). For analyses, we used a binary cumulative score of 0 ACEs versus 1 or more ACEs.

##### Household Factors

Parents reported on their own physical health and mental health status. The response options were “(1) excellent, (2) very good, (3) good, (4) fair, or (5) poor”. For analyses, we categorized both variables, parent physical health and parent mental health to two categories, either “excellent/very good” or “good/fair/poor”.

The highest level of education completed by parents was reported. Response options were “(1) less than high school, (2) high school and some college/associates degree, and (3) college degree or higher”.

The total family income was used to measure household poverty level. This was categorized by NSCH into poverty level categories based on the USA federal poverty level: (0–99% Federal Poverty Level (FPL), 100–199% FPL, 200–399% FPL, 400% FPL or greater).

##### Community Factor

NSCH categorized geographical region of residence according to US census bureau classification: Northeast, Midwest, South, or West.

### 2.3. Statistical Analysis

All estimates accounted for complex survey design and were weighted to be nationally representative of the population of children 6–17 years of age in the US. Analyses were conducted with Stata version 14.2 (StataCorp, College Station, TX, USA) [38]. Hypothesis testing was two-sided with α level set at 0.05. Demographic characteristics were summarized using descriptive statistics, and comparisons between youth with and without chronic pain and with and without food insufficiency were conducted using *t*-tests for continuous variables and chi-squared tests for categorical variables.

To address the study’s objectives, we first estimated prevalence rates of chronic pain by each level of food insufficiency (food-sufficient, mild food insufficiency, and moderate/severe food insufficiency). We also estimated prevalence rates of chronic pain (yes/no) with each of the specified covariates. We then tested the associations between food insufficiency (primary independent variable) and chronic pain (primary dependent variable) using a multivariable logistic regression model controlling for specified covariates. The covariates were individual factors (age, biological sex, race/ethnicity, anxiety, depression, any other chronic health conditions, ACEs), household factors (parent mental health, parent physical health, poverty category, parent education), and community factor (geographic region of residence).

## 3. Results

### 3.1. Demographic and Clinical Characteristics

Our sample included 48,410 children from 6 to 17 years of age, who represented a population of 45 million children. In survey-weighted analysis, 51.1% were males (Table 1). Children were predominantly Hispanic (25.4%) or non-Hispanic White (50.9%). Overall, 10.9% reported symptoms of anxiety and 4.9% of depression, while more than one-third (35.5%) of children had other chronic health conditions. A high proportion (46.2%) endorsed one or more ACEs. In this sample, 17% of children resided 0–99% below the FPL, and over 60% resided 200% or above the FPL. Almost half of parents had a college degree or higher (49.5%). Nearly all parents (>90%) reported that their physical and/or mental health was “very good/excellent”.

Greater than a quarter of children (26.1%; weighted estimate = 12 million) lived in households with mild food insufficiency, while an additional 5.1% (weighted estimate = 2.5 million) lived in households with moderate/severe food insufficiency. The overall prevalence of chronic pain in our full sample was 9.2% (weighted estimate = 4.4 million). Prevalence of chronic pain by individual, household, and community factors are presented in Table 2. As expected, there was a higher prevalence of chronic pain in older children, females, those with anxiety, depression, chronic health conditions, or who had experienced ACEs (*p* ≤ 0.001). The prevalence of chronic pain was also higher for children whose parents reported poorer mental and physical health (*p* = 0.047; *p* < 0.001).

### 3.2. Prevalence of Chronic Pain by Household Food Insufficiency Status

As hypothesized, the prevalence of chronic pain was significantly higher among children with food insufficiency compared to those who were food-sufficient (*p* < 0.0001). For children living in food-sufficient households, the prevalence of chronic pain was 6.7% (95% CI: 6.1–7.3). In contrast, 13.7% (95% CI: 12.3–15.2) of children with mild food insufficiency experienced chronic pain, with an even higher prevalence at 20.6% (95% CI: 16.3–25.6) for children with moderate/severe food insufficiency (Table 2).

### 3.3. Adjusted Associations between Food Insufficiency and Chronic Pain, Controlling for Individual, Household, and Community Covariates

Results from the multivariable logistic regression analysis revealed that after adjusting for individual, household, and community factors, food security was associated with chronic pain (*p* < 0.0001). Children with mild food insufficiency had 1.6 times greater odds of having chronic pain (95% CI: 1.4–1.9) as compared to children living in food-sufficient households. Similarly, for children with moderate/severe food insufficiency, there was 1.9 times greater odds of having chronic pain (95% CI: 1.4–2.7).

## 4. Discussion

In this secondary data analysis of a nationally representative sample of children ages 6–17 years across the US, we found that food insufficiency was strongly associated with increased prevalence of chronic pain in both unadjusted and adjusted analyses. Specifically, 13.7% to 20.6% of children living with food insufficiency had chronic pain, compared to only 6.7% of children who resided in food-sufficient households. After controlling for individual-, household-, and community-level covariates, children living with food insufficiency had 1.6 to 1.9 times the odds of having chronic pain compared to children who were food-sufficient. This pattern of findings is consistent with our hypotheses as well as prior studies that examined associations between food insufficiency and chronic pain [25,26,27,39,40].

In this sample, the prevalence of chronic pain was 6.7%. The data are varied in the literature, ranging from 6% to 57%, likely due to differences in measurement, such as the reporting period, duration, and frequency of pain symptoms assessed, and study design [3]. In this study, children were categorized as having chronic pain when parents endorsed that their child had “frequent or chronic difficulty with repeated or chronic physical pain during the past 12 months”. This likely identified a cohort of children with pain symptoms of greater pain severity and longer duration, in addition to pain-related functional impairment. Childhood chronic pain is associated with important long-term negative consequences, including symptom persistence into adulthood, increased risks for poorer physical and mental wellbeing, and socioeconomic disparities as adults [41,42,43]. Chronic pain remains underrecognized and undertreated in children, particularly for those who identified as from minoritized racial/ethnic groups and with lower socioeconomic status [44,45]. In addition, youth with chronic pain also face barriers to care, particularly for specialty pain clinic services [46]. In the context of food insufficiency, the competing priorities of procuring food and pursuing evaluation and treatment for chronic pain may be further hindered. Therefore, it is critical that our healthcare systems direct efforts to reduce barriers for these at-risk children [47].

Food insufficiency and food insecurity has long been recognized as a complex, dynamic, and multidimensional phenomenon [48], and there are several hypothesized mechanisms that may underlie its relationship with chronic pain. The conceptual framework for food insecurity and chronic disease proposed by Weiser suggests several potential avenues for further basic and clinical research in this area [35]. For example, irregular dietary patterns are more common in children from food-insecure households, which have also been identified as a risk factor for the development of recurrent headaches and abdominal pain [49,50]. Next, there are shared psychosocial risk factors that have been shown to predict the onset and persistence of food insecurity and chronic pain. For example, exposure to chronic stress is more common in children from food-insecure households and has been linked to epigenetic, hormonal, and immune functions that are known to increase risk for the development of chronic pain [51,52,53]. Moreover, food insufficiency has been found to increase the risk of sleep disturbance, a recognized predictor of chronic pain conditions [54,55]. At the household level, food insecurity has causes rooted in economics, whereby paying for food competes with pursuing medical treatments for acutely painful conditions (e.g., musculoskeletal injuries, post-surgical pain), thereby increasing the risk for the transition from acute to chronic pain [56]. Finally, food insecurity and chronic pain may be linked through broader community factors, such as geographic region of residence. National surveys have demonstrated shared patterns in the geographic variation and overlap in the US for both food insecurity and chronic pain [57,58].

The AAP has recommended that all children should be screened for household food insecurity during routine healthcare visits [16], and brief validated screening tools are available, such as the practical two-item screening tool developed by Hager et al. [59]. However, research shows that less than 30% of physician and hospital-based practices routinely screen youth for food insecurity [60]. One important consideration is that families with food insecurity may experience barriers to healthcare access, limiting the efficacy of healthcare-practice-based screening strategies. Difficulties accessing care for both routine and urgent issues, postponed well-child visits, and deferred prescribed medications have been documented in households with food insecurity [61,62]. For this vulnerable group, a multiprong approach is needed. In addition to healthcare-driven approaches, school- and community-based initiatives can identify and refer youth at risk for food insecurity to state and federal food-assistance programs (e.g., Supplemental Nutrition Assistance Program, the Summer Food Program), shown to reduce food insufficiency and insecurity, improve health outcomes, and lower healthcare costs [63,64,65]. It is critical that these initiatives are well implemented to enhance the success of these programs. Most recently, the coronavirus pandemic has unmasked numerous existing vulnerabilities in food systems and revealed deep injustices for populations from lower socioeconomic strata and/or who experience systemic racism [66]. Our findings highlight the importance of medical- and community-based initiatives to address food security as a fundamental necessity, and to understand its downstream implications on the health and wellbeing of children with highly prevalent medical conditions such as chronic pain.

Findings from this study should be assessed in light of the following limitations. First, measurement of chronic pain was based on a single-item parent-report of child pain frequency over the prior 12 months. Although we found that the prevalence of chronic pain in this sample was lower, the findings remained consistent with published data [1]. The measurement of food insufficiency was also based on a single item. Although single-item measurements of food insufficiency have demonstrated face validity and external validity [67], comprehensive measures such as the United States Department of Agriculture Food Security Survey Module should be considered in future studies. Given the cross-sectional study design, causal pathways between food insufficiency and chronic pain cannot be established. This relationship may be bidirectional; with food insufficiency acting as a chronic stressor that precedes the development of chronic pain, and/or living with pain negatively impacts the household’s psychological and financial resources, which threatens the ability to procure adequate and nutritious food. Our findings provide support for future studies to examine longitudinal and bidirectional trajectories of food insufficiency and chronic pain development and persistence in the pediatric population. Finally, findings from this study are based on a nationally representative sample of children in the community, and may not generalize to clinical samples (e.g., youth with chronic pain receiving care in a pediatric pain clinic).

## 5. Conclusions

In summary, our findings demonstrate a dose–response relationship between food insufficiency and childhood chronic pain in a nationally representative sample of US children. There is an urgent need to implement surveillance and policy remedies to negate the consequential impact of food insufficiency on health and wellbeing. In particular, for children with both food insufficiency and chronic pain, the risks of poor outcomes may be further magnified.

## Figures and Tables

**Table 1:** Sociodemographic and clinical characteristics of study sample (N = 48,410).

Characteristics		n	%	95% CI
Chronic pain	No	43,999	90.8	(90.2–91.4)
	Yes	4411	9.2	(8.6–9.8)
Food insufficiency status	Food-sufficient	35,735	68.8	(67.8–69.7)
	Food-insufficient: mild	11,026	26.1	(25.2–27.0)
	Food-insufficient: moderate/severe	1649	5.1	(4.6–5.7)
Age categories	6–11 years	24,590	58	(57.0–59.0)
	12–17 years	23,820	42	(41.0–43.0)
Biological sex of child	Male	25,057	51.1	(50.1–52.1)
	Female	23,353	48.9	(47.9–49.9)
Race and ethnicity	non-Hispanic, White	33,042	50.9	(49.9–52.0)
	non-Hispanic, Black/African American	3181	13.1	(12.4–13.9)
	Hispanic	6010	25.4	(24.2–26.5)
	non-Hispanic, Asian	2540	4.6	(4.2–5.0)
	non-Hispanic, other	3637	6	(5.6–6.4)
Anxiety	No	41,454	89.1	(88.5–89.6)
	Yes	6595	10.9	(10.4–11.5)
Depression	No	45,330	95.1	(94.6–95.5)
	Yes	2901	4.9	(4.5–5.4)
Any other chronic health conditions ^a^	No	29,230	64.5	(63.6–65.4)
	Yes	19,180	35.5	(34.6–36.4)
Any ACEs	No	27,179	53.8	(52.8–54.8)
	Yes	21,228	46.2	(45.2–47.2)
Parent mental health	Very good-excellent	45,793	94.4	(93.9–94.8)
	Good-fair-poor	2617	5.6	(5.2–6.1)
Parent physical health	Very good-excellent	45,156	92	(91.3–92.6)
	Good-fair-poor	3125	8	(7.4–8.7)
Poverty category	0–99% FPL	5478	17	(16.2–17.9)
	100–199% FPL	7961	21.9	(21.0–22.9)
	200–399% FPL	15,038	29.8	(28.8–30.7)
	400% FPL or greater	19,933	31.3	(30.5–32.2)
Parent education	Less than High School	1241	9.9	(9.0–10.9)
	High School and some college/associates degree	17,778	40.5	(39.5–41.5)
	College degree or higher	29,391	49.5	(48.5–50.5)
Geographic region of residence	Northeast	8198	15.5	(14.9–16.2)
	Midwest	11,238	21.2	(20.6–21.9)
	South	15,199	38.6	(37.6–39.6)
	West	13,775	24.7	(23.6–25.7)

CI: confidence interval; N: total sample of 48,410 children, representing an estimated population of 45 million children. %: Percentages were obtained from survey-weighted analysis; a. chronic health conditions included asthma, attention-deficit and hyperactivity disorder, allergies, autism, diabetes mellitus, neurodevelopmental delay, arthritis, cystic fibrosis, seizures, and heart conditions; ACEs: adverse childhood experiences; FPL: Federal Poverty Level.

**Table 2:** Sociodemographic correlates of chronic pain: multivariate logistic regression adjusted for individual, household, and community factors.

		Chronic Pain				
		Yes (%)	95% CI	Adj. OR	95%CI	*p*
Food insufficiency status	Food-sufficient	6.7	6.1–7.3	Ref		
	Food-insufficient: mild	13.7	12.3–15.2	1.6	1.4–1.9	<0.0001
	Food-insufficient: moderate/severe	20.6	16.3–25.6	1.9	1.4–2.7	<0.0001
Age categories	6–11 years	7.1	6.3–7.9	Ref		
	12–17 years	12.1	11.3–13.1	1.5	1.3–1.8	<0.0001
Biological sex of child	Male	7.6	7.0–8.4	Ref		
	Female	10.9	9.9–11.9	1.6	1.4–1.9	<0.0001
Race and ethnicity	non-Hispanic, White	8.6	8.1–9.1	Ref		
	Hispanic	9.5	8.0–11.3	0.9	0.7–1.2	0.43
	non-Hispanic, Asian	11.1	9.3–13.1	1.2	1.0–1.5	0.053
	non-Hispanic, Black/African American	4.9	3.4–7.1	0.8	0.5–1.2	0.267
	non-Hispanic, other	9.4	7.6–11.5	1.0	0.7–1.2	0.725
Anxiety	No	7.3	6.7–8.0	Ref		
	Yes	24.5	22.2–26.9	1.9	1.5–2.3	<0.0001
Depression	No	7.9	7.4–8.5	Ref		
	Yes	33.9	29.5–38.8	2.1	1.5–2.8	<0.0001
Any other chronic health conditions ^a^	No	6.1	5.5–6.8	Ref		
	Yes	14.8	13.7–16.1	2.2	1.9–2.6	<0.0001
Any ACEs	No	6.0	5.4–6.7	Ref		
	Yes	12.9	11.9–14.0	1.3	1.1–1.6	0.001
Parent mental health	Very good-excellent-good	8.4	7.9–9.1	Ref		
	Good-fair-poor	22.2	18.5–26.3	1.4	1.0–1.9	0.047
Parent physical health	Very good-excellent-good	8.2	7.7–8.9	Ref		
	Good-fair-poor	20.7	17.6–24.2	1.6	1.2–2.0	0.001
Poverty category	0–99% FPL	12.3	10.5–14.3	Ref		
	100–199% FPL	11.1	9.7–12.7	1.0	0.8–1.3	0.931
	200–399% FPL	8.5	7.6–9.6	0.9	0.7–1.2	0.489
	400% FPL or greater	6.9	6.1–7.8	1.0	0.7–1.2	0.745
Parent education	Less than high school	11.7	8.9–15.3	Ref		
	High school and some college/associates degree	10.7	9.8–11.6	0.8	0.6–1.2	0.339
	College degree or higher	7.5	6.8–8.3	0.8	0.6–1.1	0.21
Geographic region of residence	Northeast	8.3	7.2–9.6	Ref		
	Midwest	8.6	7.8–9.6	1.0	0.8–1.2	0.988
	South	9.5	8.5–10.5	1.1	0.9–1.3	0.479
	West	9.9	8.4–11.6	1.2	1.0–1.6	0.08

CI: confidence interval; N: total sample of 48,410 children, representing an estimated population of 45 million children. %: Percentages were obtained from survey-weighted analysis; a. chronic health conditions included asthma, attention-deficit and hyperactivity disorder (ADHD), allergies, autism, diabetes mellitus, neurodevelopmental delay, arthritis, cystic fibrosis, seizures, and heart conditions; Adj. OR: adjusted odds ratio; FPL: Federal Poverty Level; Ref: reference.

## Data Availability

The data presented in this study are available on request from the corresponding author.
